# Dietary fiber and probiotics for the treatment of atypical antipsychotic-induced metabolic side effects: study protocol for a randomized, double-blind, placebo-controlled trial

**DOI:** 10.1186/s13063-021-05123-w

**Published:** 2021-02-23

**Authors:** Chenchen Liu, Dongyu Kang, Jingmei Xiao, Yuyan Huang, Xingjie Peng, Weiyan Wang, Peng Xie, Ye Yang, Jingping Zhao, Renrong Wu

**Affiliations:** 1 National Clinical Research Center for Mental Disorders, and Department of Psychiatry, The Second Xiangya Hospital of Central South University, Changsha, 410011 Hunan China; 2 Shanghai Institutes for Biological Sciences, Chinese Academy of Sciences, Shanghai, 200031 China

**Keywords:** Dietary fiber, Probiotics, Atypical antipsychotic medications, Metabolic side effects

## Abstract

**Background:**

Atypical antipsychotic medications, which are effective for the treatment of schizophrenia and bipolar disorder, are associated with features of metabolic syndrome, such as weight gain, hyperglycemia, dyslipidemia, and insulin resistance. Although there are a few studies on the effects of dietary fiber or probiotics on weight loss in obese people, no published trials have reported the efficacy of dietary fiber and probiotics on reducing atypical antipsychotic-induced weight gain.

**Methods:**

For this 12-week randomized, double-blind, placebo-controlled study, 100 patients with a weight gain of more than 10% after taking atypical antipsychotic medications were recruited. Participants were randomized to four groups as follows: probiotics (840 mg twice daily (bid)) plus dietary fiber (30 g bid), probiotics (840 mg bid) plus placebo, placebo plus dietary fiber (30 g bid), or placebo group. The primary outcome was the change in body weight. Secondary outcomes included changes in metabolic syndrome parameters, appetite score, biomarkers associated with a change in weight, and gut microbiota composition and function.

**Discussion:**

To date, this is the first randomized, placebo-controlled, double-blinded trial investigating the efficacy of dietary fiber and probiotics alone and in combination to reduce metabolic side effects induced by atypical antipsychotic medications. If effective, it is possible to conclude that dietary fiber and probiotics can reduce atypical antipsychotic-induced metabolic side effects.

**Trial registration number:**

ClinicalTrials.gov NCT03379597. Registered on 19 November 2017.

**Supplementary Information:**

The online version contains supplementary material available at 10.1186/s13063-021-05123-w.

## Background

Atypical antipsychotic medications are widely used to treat patients with psychiatric disorders, such as schizophrenia and bipolar disorder. To control such conditions and prevent relapse, most patients must take antipsychotic medications for a long time or even their entire life. However, almost all atypical antipsychotic medications can cause metabolic side effects, such as weight gain, hyperglycemia, dyslipidemia, and insulin resistance [1–5]. For example, the long-term use of these drugs, especially olanzapine and clozapine, can cause various metabolic side effects in 50% of patients [6]. Alvarez-Jiménez et al. confirmed that 78.8% of patients treated with antipsychotic medications gained weight by more than 7% [7]. Metabolic side effects not only reduce the patient’s adherence to treatment but also affect morbidity and mortality [8]. Some researchers [9, 10] have suggested that the life expectancy for people with severe mental illness is 30 years shorter than the average, and that weight gain is one of the major risk factors for heart disease and early death in patients on antipsychotics. Therefore, it is urgent to explore potential interventions for metabolic side effects related to atypical antipsychotic medications.

To date, the mechanisms underlying metabolic side effects caused by atypical antipsychotic medications are not clear, resulting in a lack of effective treatments. In recent years, with a rise in gut microbiota research, it has been proposed that atypical antipsychotic-induced weight gain is mediated by changes in the gut microbiota composition [11]. The gut microbiota plays an important role in metabolic diseases and energy balance [12]. It increases the levels of hormones closely related to appetite and glucose tolerance, such as peptide YY (PYY), ghrelin, insulin, and glucagon-like peptide-1 (GLP-1) which enhance the absorption of short-chain fatty acids (SCFAs) [13]. In addition, various microbial groups are thought to affect hormonal signaling pathways, including leptin (LP) and ghrelin [13], and are thought to play a role in regulating host epigenetics [14]. Recently, Chen et al. showed that the LP: adiponectin (LP/ADPN) ratio might be a key indicator for atypical antipsychotic-induced metabolic syndrome [15]. Changes in the structure of the gut microbiota will increase the level of lipopolysaccharide (LPS), leading to overfeeding and metabolic side effects [16]. Consequently, improving the gut microbiota might be beneficial for atypical antipsychotic-induced metabolic side effects.

Yadav et al. showed that probiotics, comprising living microorganisms, can effectively reduce obesity and diabetes in mouse models by regulating the gut microbiota [17]. *Bifidobacterium* can reduce weight gain and adipose tissue in obese rats fed a high-fat diet [18–20]. Furthermore, probiotics exert a prophylactic effect on obesity by regulating the gut microbiota to reduce LPS production [21]. As an indigestible polysaccharide, dietary fiber can promote the growth of probiotics when obtained from the diet [22]. Kao et al. showed that intake of the dietary fiber galacto-oligosaccharide (B-GOS) significantly reduces olanzapine-induced weight gain, indicating that B-GOS can serve as a preventive treatment [23]. Moreover, in a meta-analysis of dietary fiber and probiotics with respect to regulation of the gut microbiota to fight obesity, it was found that probiotics could promote the growth of bifidobacteria and effectively alleviate obesity [24]. Therefore, it is possible that dietary fiber and probiotics might have benefits with respect to metabolic side effects associated with atypical antipsychotic medications. In our previous study on probiotic supplements that reduce atypical antipsychotic-induced metabolic disturbances in drug-naïve first-episode schizophrenia, we found that they were effective and safe in attenuating atypical antipsychotic-induced elevations in fasting insulin and insulin resistance but not weight gain. However, to the best of our knowledge, no double-blind, placebo-controlled studies have directly compared dietary fiber and probiotics alone or in combination for metabolic side effects induced by atypical antipsychotic medications. In this protocol, a 12-week randomized, double-blind, placebo-controlled trial to test the efficacy of dietary fiber and probiotics alone and in combination to reduce atypical antipsychotic-induced metabolic side effects in patients with schizophrenia or bipolar disorders is reported in detail.

## Methods/design

### Aim

To design and establish a protocol for a 12-week randomized, double-blind, placebo-controlled trial to test the efficacy of dietary fiber and probiotics alone and in combination to reduce metabolic side effects induced by atypical antipsychotic medications in patients with schizophrenia or bipolar disorders.

### Study design

The study is a 12-week parallel, double-blind, placebo-controlled, single-center randomized controlled trial.

### Hypothesis

Our hypothesis is that a combination of dietary fiber and probiotics can improve the dysfunction of the gut microbiota and the atypical antipsychotic-induced metabolic side effects.

### Study setting

Patients treated in the schizophrenia or bipolar disorder outpatient clinic of the Mental Health Institute of the Second Xiangya Hospital, Central South University, China, between August 2019 and May 2021, will be recruited.

### Inclusion criteria

The inclusion criteria were as follows:

1. Patients aged 18–45 years with schizophrenia or bipolar disorder diagnosed according to the criteria set out in the *Diagnostic and Statistical Manual of Mental Disorders Fifth Edition (DSM-5).*
2. Patients with relatively stable improvement, as well as a total Positive and Negative Syndrome Scale (PANSS) score of ≤ 60 for patients with schizophrenia or a total Hamilton Depression Scale, also called HAMD-17, score of ≤ 7, and a total Young Mania Rating Scale (YMRS) score of < 5 for patients with bipolar disorder.
3. Patients with a weight gain of more than 10% after taking atypical antipsychotic medications.
4. All participants cared for by their parents or other adult caregivers who could monitor and record their dietary fiber, probiotics, and atypical antipsychotic medication intake each day of the trial to monitor adherence.
5. Participants without problems such as relocation of residence, inconvenience of transportation, and difficulty visiting the clinic during the entire research process.
6. Participants without a history of allergy to dietary fiber and probiotics.
7. An informed consent signed by patients and guardians.
8. Atypical antipsychotic medications that remained at a fixed dose throughout the course of treatment. Only trihexyphenidyl for extrapyramidal symptoms and lorazepam for insomnia or agitation could be used additionally (when necessary).

### Exclusion criteria

The exclusion criteria were as follows:

1. Intake of psychoactive substances or substance abusers.
2. Patients with a severe physical disability who could not complete follow-up.
3. Patients with other severe mental illness, intellectual disability, dementia, and severe cognitive impairment, who met the diagnostic criteria of DSM-5.
4. Patients who were suicidal or uncooperative.
5. Patients who were enrolled in or preparing to enroll in other clinical studies.
6. Patients who used antibiotics in the 2 months prior to this study, as well as fiber supplements, microecological live bacteria, or laxatives, such as polyethene glycol, in the last 6 weeks.
7. Patients with a history of gastrointestinal diseases, such as uncontrolled and recurrent diarrhea, active peptic ulcer, or gastrointestinal bleeding.
8. Taking other medications that contribute to weight gain.

### Intervention, randomization, allocation, and blinding

Eligible participants were randomized into four groups in a balanced 2 × 2 factorial design for 12 weeks as follows: probiotics (Bifico, triple live bacteria oral capsule, 840 mg twice daily (bid)) plus dietary fiber (Perfect, 10 g HI-FIBER DRINK plus 20 g Extra Herb Powder, 30 g bid), probiotics (840 mg bid) plus dietary fiber placebo, probiotics (30 g bid) placebo plus dietary fiber, or probiotics placebo plus dietary fiber placebo. The choice of dietary fiber was based on the results of the research by Zhao et al. [25] The choice of maltodextrin as a dietary fiber placebo was based on the results of Vandeputte et al. [26]. Bifico (Shanghai Xinyi Pharmaceutical Inc., Shanghai, China) is a commonly used probiotic supplement in China containing a combination of live *Bifidobacterium*, *Lactobacillus*, and *Enterococcus* bacteria at a concentration of at least 5.0 × 10^7^ CFU/g. In addition, Perfect dietary fiber (Perfect Co., Ltd., Jiangsu, China) is a commonly used supplement in China. Participants were required to implement the diet naturally throughout the trial.

Participants were randomized through a computer-generated table in blocks of eight to ensure approximately equal numbers of participants in each group. The distribution sequence was randomly generated by the research assistant based on a computer-based pre-generated intervention order. The assignment was determined after patients completed all screening assessments and were accepted into the study.

During the study, all researchers and subjects were blinded. After purchasing from the manufacturers, all dietary fiber and maltodextrin were divided into opaque sealed bags, and the labels on the bottles of all Bifico capsules and placebo capsules were torn off by a research assistant and relabeled. The acceptability and adverse events associated with the study products were monitored. If needed, discontinuation or modification of the treatment was considered at the discretion of the physician. Unblinding occurred after the final data analysis. After the double-blind treatment phase, participants were encouraged to enter a 12-week follow-up phase. No study medicine would be administered during this phase. At the start of the follow-up phase, further clinical treatment would be arranged by the study investigator and/or the subjects’ treating physician.

The participants’ adherence to probiotics and dietary fiber treatment for each visit was defined as taking more than 80% of the study drug dosage prescribed for that interval. The importance of taking the prescribed amount of study medication was explained when a participant failed to adhere. To ensure participant adherence, participants received a call every week. Face-to-face adherence discussions were held on the first visit, and the importance of research guidelines and research product instructions were emphasized in each subsequent research visit. Participants were required to bring all the remaining capsules and sealed bags to each visit. To improve the validity of the data, capsules and sealed bags were counted at each study visit, and the percent participant adherence to the treatment was calculated according to the number of capsules and sealed bags taken and the expected intake.

### Outcomes

#### Primary

The primary outcome measure was the change in body weight between baseline and week 12 after treatment.

#### Secondary

Secondary outcome measures were as follows:

1. Body mass index, calculated as weight in kilograms divided by height in meters squared, waist circumference, and hip circumference.
2. Fasting glucose; fasting insulin level; insulin resistance index (IRI), which is calculated according to the following formula: fasting insulin (mIU/L) × fasting glucose (mmol/L)/22.5; blood counts; liver and renal function; lipids, which include triglycerides, total cholesterol, high-density lipoprotein, and low-density lipoprotein, and glycated hemoglobin (HbA1c).
3. Biomarkers associated with changes in weight (GLP-1, LP, ghrelin, ADPN, LPS, and PYY).
4. Changes in appetite; a self-reported scale was used to record the percentage change in appetite at each follow-up visit.
5. Differences in gut microbiota composition (bacterial relative abundance and diversity) and function (stool SCFA concentrations and relative abundances of functional bacterial genes) between baseline and the endpoint.
6. The PANSS was used to measure the positive and negative symptoms of schizophrenia. The YMRS and HAMD were used to measure mania and depression symptoms of bipolar disorder. The Treatment Emergent Symptom Scale (TESS) was used to assess adverse reactions in patients taking atypical antipsychotic medications.

### Trial visits, assessments, and outcome measures

**Fig. 1:**
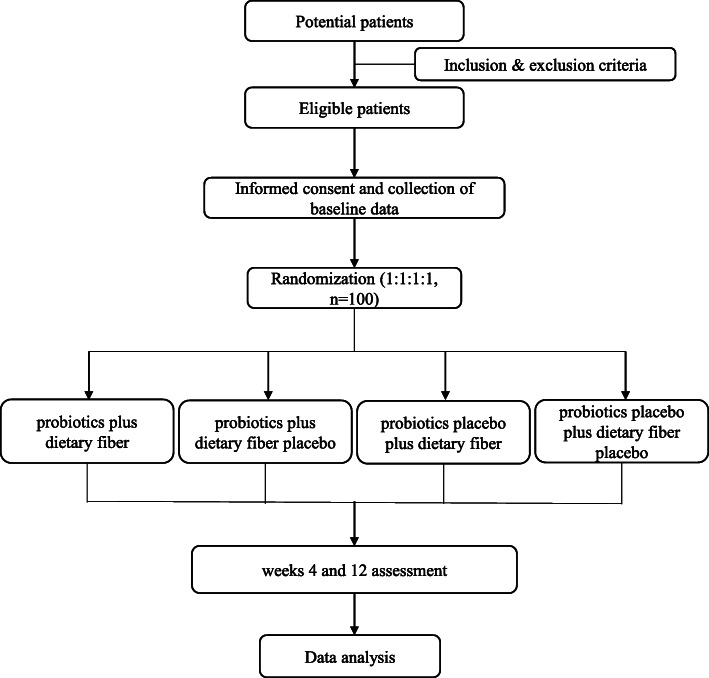
Flow chart of participants from screening to follow-up

**Fig. 2:**
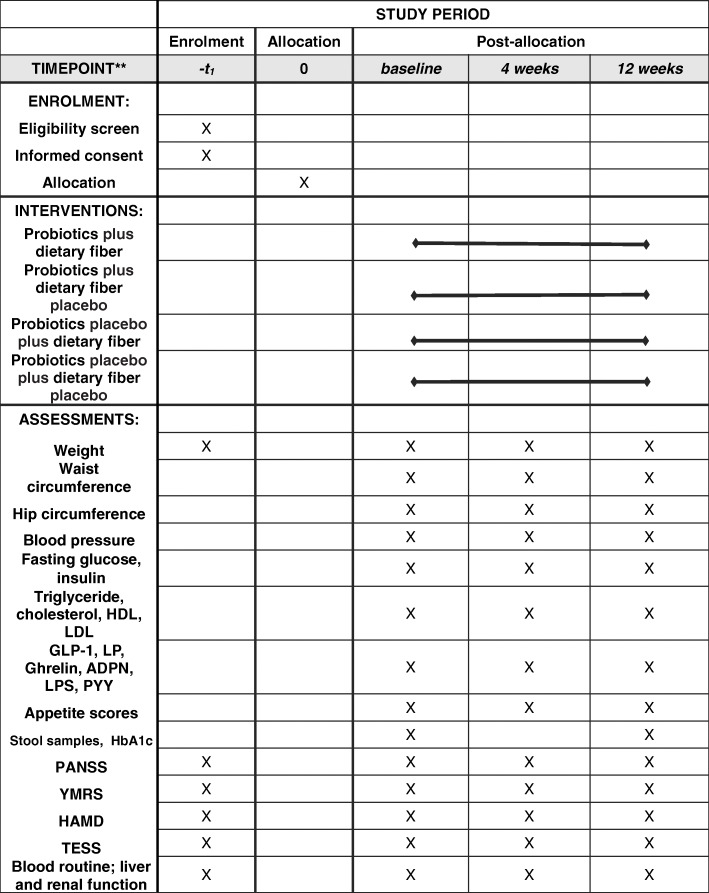
Standard Protocol Items: Recommendations for Interventional Trials (SPIRIT) schedule for enrollment, treatment, and assessments

Figure 1 shows the flow of participants from screening to follow-up. The participants were followed up at weeks 0, 4, and 12. At all visits, adverse events and current medication were recorded. The study products were dispensed at every study visit. At screening (visit 0), demography, medical history, and concomitant medication were recorded. Participants had three blood tests (fasting glucose, fasting insulin, routine blood, including liver and renal function, lipids, GLP-1, LP, ghrelin, ADPN, LPS, and PYY) at baseline and weeks 4 and 12. Participants were evaluated on the PANSS, YMRS, HAMD, TESS, appetite score scales and weight, waist circumference, hip circumference, blood pressure, and electrocardiograms were measured at baseline and at the 4th and 12th weeks. HbA1c and stool samples were obtained from participants at baseline and weeks 12. Study visits and assessments are presented in Fig. 2.

All assessments were conducted by trained members of the research team. All anthropometric measurements were collected by the research team; participants were asked to wear light clothing, remove their shoes, and empty their bladders. Height was recorded at baseline. At each visit, the weight was recorded to the nearest 0.1 kg using calibrated scales. Waist circumference was measured in the horizontal plane to the nearest 0.5 cm using a non-stretchable measuring tape placed around the abdomen at a level halfway between the top of the iliac crest and the bottom of the ribs. Hip circumference was measured at the maximum circumference of the buttocks [27]. Blood pressure was measured with a manometer (cuff in two-thirds of the upper right arm) after 10 min of resting in a sitting position at the beginning and end of the study. The collection of stool samples required participants to (1) urinate cleanly to prevent contaminated stool, (2) open their bowels on to a clean bedpan, (3) use a sterile sampling spoon to remove the surface layer of feces, (4) take out about 3–4 g of middle feces, (5) put in a sterile container, and (6) repeat sampling three times. The specimen container was immediately sent to the laboratory for freezing and stored at − 80 °C.

### Sample size calculations

The primary endpoint is the difference in body weight between groups. According to Parnell and Reimer [28], the value of the mean difference can be assumed as 2, with a standard deviation (SD) of 0.2. To detect this difference, based on a power of 90%, a significance level of 5%, and 20% of patients lost to follow-up, it was calculated that 25 patients were needed for each group. The sample size calculation was conducted using Power Analysis and Sample Size statistical software (11.0.7).

### Data analysis

All analyses were based on an intention-to-treat approach (or strategy), which included all randomized patients, and the results were made available (including dropouts and withdrawals). All analyses were conducted using the Statistical Package for Social Sciences, version 22 (SPSS Inc., Chicago, IL). Continuous variables were described using means and 95% confidence intervals (CIs). Categorical variables were described using frequencies and percentages. Data normality was examined using a Kolmogorov-Smirnov test. Normally distributed variables are presented as the mean ± SD. Non-parametric statistics and appropriate log-transformation were performed if the assumption of normality was not met. A two-tailed *p* value of less than 0.05 was considered statistically significant. Hypothesis testing methods *t* test, *χ*^2^ analysis, and analysis of variance were used as appropriate. A CI of 95% was considered in all tests.

### Involvement of patients and the public

Before recruitment, with some outpatients with schizophrenia and bipolar disorder the feasibility of the study design and interventions were discussed, based on which the number and time of follow-ups were adjusted properly. No patients or the public participated in the recruitment and evaluation of the results. The results of the study were sent to participants in the form of a written report and email. During the experiment, the enrolled patients were unaware of the intervention products.

### Ethics and dissemination

Following and based on the *Helsinki Declaration*, this study was approved by the Ethics Committee of the Second Xiangya Hospital of Central South University. All participants provided signed informed consent before becoming involved in this study and could withdraw at any phase without giving a reason. The adverse events were continuously monitored; participants were asked to report any problems throughout their participation in the study. We also asked participants if they had any discomfort weekly. An adverse event assessment was conducted to determine whether to terminate the study. The probiotic placebo was purchased from the manufacturer of Bifico. The maltodextrin was purchased from the Qianzhi food manufacturer. And we declared no conflict of interest with the manufacturer.

The findings of the study will be disseminated through various approaches, including peer-reviewed journal publications, and international conference presentations. This protocol was designed according to the Standard Protocol Items: Recommendations for Interventional Trials (SPIRIT) guidelines [29] (see Additional file 1).

### Data management

The research team ensured data confidentiality. The collected data was stored in the Doctor-Patient Cloud Bridge Database (https://research.fulcruminfo.cn/#/login?url=%2Fhome), which is protected by a password, and the research team was the only party able to access the database. The password was changed regularly. After the study, all data in the database was downloaded and stored on a password-protected computer before being deleted from the database.

## Discussion

Atypical antipsychotic medications are important drugs for schizophrenia and bipolar disorders, but their metabolic side effects are still a problem for many patients and doctors. Many studies have shown that dietary fiber and probiotics can effectively reduce weight and improve the metabolism in obese people by improving the gut microbiota [30], but there are few studies on dietary fiber and probiotics acting on atypical antipsychotic-induced metabolic side effects. To date, there have been no randomized, double-blind, controlled trials to study the clinical feasibility of adding dietary fiber and probiotics to treat these metabolic side effects. Therefore, research evidence is urgently needed to prove that dietary fiber and probiotics can be used for this purpose.

This is the first protocol describing a randomized, double-blind, placebo-controlled clinical trial to evaluate the clinical feasibility of dietary fiber and probiotics independently or in combination to treat metabolic side effects caused by atypical antipsychotic medications. The trial provided evidence of the effectiveness and safety of dietary fiber and probiotics used alone or in combination to treat atypical antipsychotic-induced metabolic side effects. The limitation of this study is that it was a 12-week trial. Therefore, it is unclear whether the improvement in metabolic indicators would continue after patients stopped taking dietary fiber and/or probiotics. In addition, appetite assessments based on self-reports were possibly subjective.

## Trial status

Protocol version 2, 23 April 2019.

Recruitment began on 12 August 2019 and approximately will end on 31 May 2021.

## Supplementary Information

**Additional file 1.** SPIRIT 2013 Checklist: Recommended items to address in a clinical trial protocol and related documents*.

## Data Availability

The results will not be available before publishing.

## Abbreviations

PYY: Peptide YY
GLP-1: Glucagon-like peptide-1
LP: Leptin
ADPN: Adiponectin
LPS: Lipopolysaccharide
B-GOS: Galacto-oligosaccharides
DSM-5: Diagnostic and Statistical Manual of Mental Disorders Fifth Edition
PANSS: Positive and Negative Syndrome Scale
HAMD: Hamilton Depression Scale
YMRS: Young Mania Rating Scale
IRI: Insulin resistance index
HbA1c: Glycated hemoglobin
SCFA: Short-chain fatty acids
TESS: Treatment Emergent Symptom Scale
SPSS: Statistical Package for Social Sciences
SPIRIT: Standard Protocol Items: Recommendations for Interventional Trials
